# Total hip arthroplasty for failed internal fixation of femoral neck fracture: a retrospective study with 2–14 years’ follow-up of 345 patients

**DOI:** 10.1186/s13018-023-03827-0

**Published:** 2023-05-09

**Authors:** Hanpeng Lu, Niu Zhu, Tingxian Ling, Jian Cao, Hong Xu, Kai Zhou, Enze Zhao, Zongke Zhou

**Affiliations:** 1 Department of Orthopaedic Surgery, West China Hospital, Sichuan University, No.37, Guoxue Road, Wuhou District, Chengdu, 610041 Sichuan People’s Republic of China; 2 Precision Medicine Center, West China Hospital, Sichuan University, No.37, Guoxue Road, Wuhou District, Chengdu, 610041 Sichuan People’s Republic of China

**Keywords:** Salvage total hip arthroplasty, Femoral neck fracture, Failed internal fixation, Clinical outcomes, Young patients

## Abstract

**Objectives:**

The purpose of this study was to analyze mid- to long-term outcomes of total hip arthroplasty (THA) following failed internal fixation of femoral neck fracture.

**Methods:**

This study retrospectively analyzed 345 patients with femoral neck fracture who underwent THA after failure of internal fixation at our hospital between January, 2003 and December, 2019. Patients older than 55 years (n = 175) and patients no older than 55 years (n = 170) were compared in terms of complications and survival rates during follow-up, which lasted a mean of 6 years.

**Results:**

The two age groups showed similarly low incidence of complications and similarly long periods of survival without revision surgery. Only three younger patients and two older patients underwent revision surgery during follow-up. The two groups showed similarly high survival rates at the end of follow-up (> 93%). Younger patients showed significantly bettter Harris hip score at last follow-up (90.2 vs. 88.1 points, p < 0.001) without clinically significant difference, but they required THA significantly earlier after internal fixation (4.4 vs. 6.8 years, p < 0.001).

**Conclusions:**

THA after failed internal fixation of femoral neck fracture is a well tolerated and effective procedure in older and younger patients.

## Introduction

Femoral neck fracture affects 1.3–2.2 million individuals every year globally, and its incidence is expected to reach 3.9–7.3 million per year by 2050 [1]. Such fracture increases the risk of morbidity and mortality, and it makes many kinds of treatment for other diseases substantially more expensive than in the absence of fracture [2, 3]. Primary total hip arthroplasty (THA) can treat such fractures, but this requires destroying the original hip joint. Therefore, clinicians often prefer to treat such fractures, when possible, with internal fixation involving cannulated screws, dynamic hip screws, intramedullary nails, or anatomic plates. Internal fixation can resolve discomfort while preserving the original hip joint [4–7]. However, internal fixation is associated with several complications, such as fracture nonunion, avulsion of the femoral head, and traumatic osteoarthritis [ref]. In fact, up to one third of patients who undergo internal fixation may require reoperation to treat such complications, while more than 10% may require salvage THA [8–12].

Salvage THA is a more complex procedure than primary THA because of the weak and osteoporotic bone, retained hardware, and anatomical deformity [13–15]. Yang et al. [16] reported the salvage procedure is longer and involves greater risk of hip complications than the primary procedure, but there are no long-term follow-up results. Although Moon et al. [13] carried out a long-term follow-up study of salvage THA in patients with femoral neck fracture after failure of internal fixation, the sample size of patients collected is not large enough. Therefore, a large cohort study with the mid-long term follow-up study is needed to further determine the efficacy and complications of salvage THA.

Moreover, little is known about whether outcomes differ substantially between older and younger patients. Young patients make up 3% of patients with femoral neck fracture [17], and they are usually the result of high-energy violent injury [18]. Compared with the elderly, young people with good physical quality have almost no osteoporosis, but large amount of activity. We do not know the exact effect and the difference among different ages, which makes it challenging to consult for specific age group who perform THA after failure of internal fixation of femoral neck fracture.

In the present study, we aimed to investigate the mid- to long-term outcomes of salvage THA following failed internal fixation for femoral neck fracture using the largest patient cohort to date. We also compared the incidence of complications and outcomes between patients younger and older than 55 years.

## Methods

### Study design and participants

This retrospective cohort study was approved by the Regional Ethics Committee of West China Hospital, Sichuan University (approval no. 2022–1545). We reviewed the records of 1068 patients with neck fracture who were hospitalized at the Joint Surgery Center of West China Hospital from January 2003 to November 2019 for eligibility. Patients who underwent total hip replacement after the failure of internal fixation for neck fracture were included. The exclusion criteria included: infection after internal fixation, pathological fracture or other complications affecting hip function, loss during follow-up after surgery, or death unrelated to surgery.

**Fig. 1:**
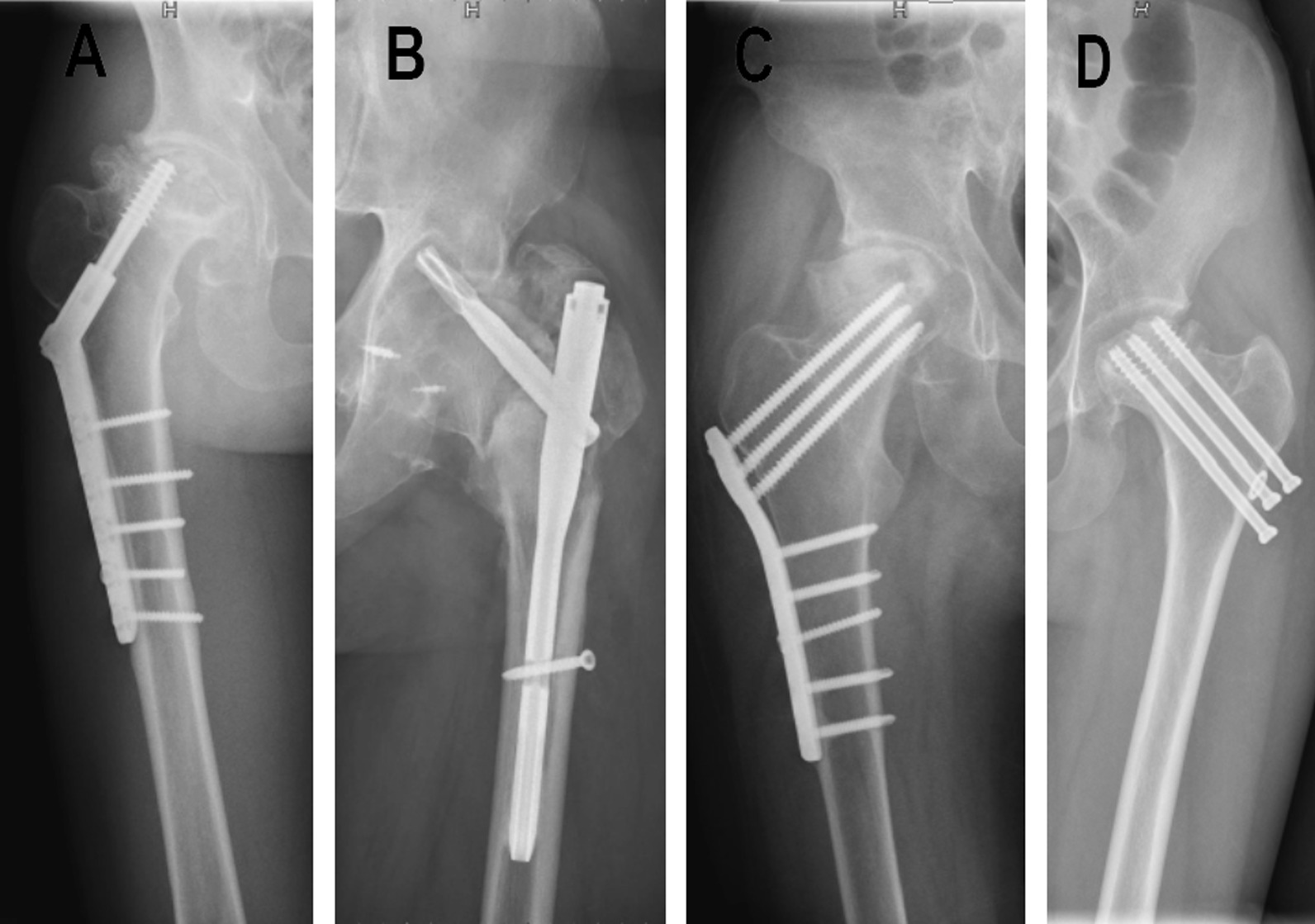
The internal fixation including dynamic hip screws (**A**), intramedullary nails (**B**), anatomical steel plates (**C**) and cannulated screws (**D**) after femoral neck fracture

The clinical, radiographic, and surgical data of the included patients were retrospectively evaluated. Internal fixation in these patients after femoral neck fracture involved intramedullary nails, dynamic hip screws, cannulated screws, and anatomical steel plates as Fig. 1 shows. The indication for THA was a failure of internal fixation after femoral neck fracture leading to femoral head necrosis, bone nonunion, or traumatic arthritis resulting in severe pain, stiffness, claudication, and difficulty in walking and daily activities.

All patients used cementless prostheses and a variety of acetabular components (size: 46#–62#) and femoral stalks (size: 8#–6#) produced by Depuy or Stryker Orthopedics Company, as detailed in the Table 1. Bearing surfaces in our research include ceramic to ceramic, ceramic to polyethylene, and ceramic to high crosslinked polyethylene selected according to the patients' situation. The operation is performed by five senior joint surgeons, who perform over 200 joint replacement operations every year, and their surgical skills are quite skilled.

**Table 1:** Implant description

Position	Implant	Company
Acetabular prosthesis	Pinnacle	DePuy Synthes
	Tridengt	Stryker
Femoral stalk	Summit	DePuy Synthes
	Trilock	DePuy Synthes
	Corail	DePuy Synthes
	AML	DePuy Synthes
	Solution	DePuy Synthes
	Accolade	Stryker

For THA, all patients underwent general anesthesia and were placed in the contralateral decubitus position through the posterolateral approach as described [16]. A longitudinal incision was made on the outside of the hip to cut through the skin, subcutaneous tissue, and joint capsule. The internal fixation was removed, then the acetabulum was shaped to prepare for the placement of acetabular prosthesis and lining. The femoral head was taken out after osteotomy, then the medullary cavity was expanded to place a femoral handle of appropriate size and the corresponding femoral head. After hip reduction, the range of motion and stability was checked in all directions, followed by repeated washing and layer-by-layer suture of the surgical incision. Following anesthesia, patients underwent a guided ankle plantar flexion/extension exercise. Patients gradually began functional exercise of quadriceps femoris in bed, walking with a walking aid, and weight-bearing walking.

All patients underwent standardized surgical procedures and perioperative management involving perioperative antibiotics, postoperative anticoagulation, ice bags on wounds, and pneumatic compression with foot pumps.

### Clinical evaluation

The medical and surgical records were reviewed for data on intraoperative blood loss, hospitalization time, 1-year postoperative mortality, all-cause reoperation, and postoperative complications. Patients attended follow-up visits at 6 weeks, 3 months, 6 months and 12 months, then once yearly thereafter. Harris hip score (HHS) was used to evaluate hip function. Postoperative complications were recorded, which included dislocation, loosening, infection, and fracture around the prosthesis.

### Radiologic evaluation

A standard radiographic assessment that included anteroposterior radiographs of the pelvis and anteroposterior and lateral radiographs of the hip was performed by a trained radiographer. The radiological evaluation was conducted by two independent observers. The locations of anteroposterior radiographs were classified according to three zones for the acetabulum and seven zones as described [19]. The loosening and osteointegration of the femoral component were evaluated in accordance with the Engh standard and were classified as bone ingrown, fibrous stable, or loose [20]. Subsidence of the femoral component was defined as described [21]. An acetabular component was considered unstable when there was a circumferential radiosclerotic line of > 2 mm in width, migration of > 2 mm based on a teardrop reference, or a change > 5° in inclination or anteversion as described [22].

### Statistical analysis

Continuous data were presented as mean and standard deviation if normally distributed, or as mean and quartile spacing if skewed, while categorical data were presented as frequency and percentage. Differences between patients older than 55 years (hereafter “older”) or those 55 years or younger (hereafter “younger”) were assessed for significance using the independent-samples Student’s *t* test in the case of normally distributed continuous data, Mann–Whitney U test in the case of skewed continuous data, or chi-squared or Fisher's exact tests in the case of categorical data.

Survival after THA was assessed using the Kaplan–Meier method, where the endpoint of survival was defined as prosthesis revision for infection, radiographic loosening, or any type of failure of prosthetic component(s). Survival curves were compared using the Mantel-Cox log-rank test.

Statistical significance was defined as two-tailed P < 0.05. All statistical analyses were performed using SPSS 28.0 (IBM, Chicago, IL, USA).

## Results

**Fig. 2:**
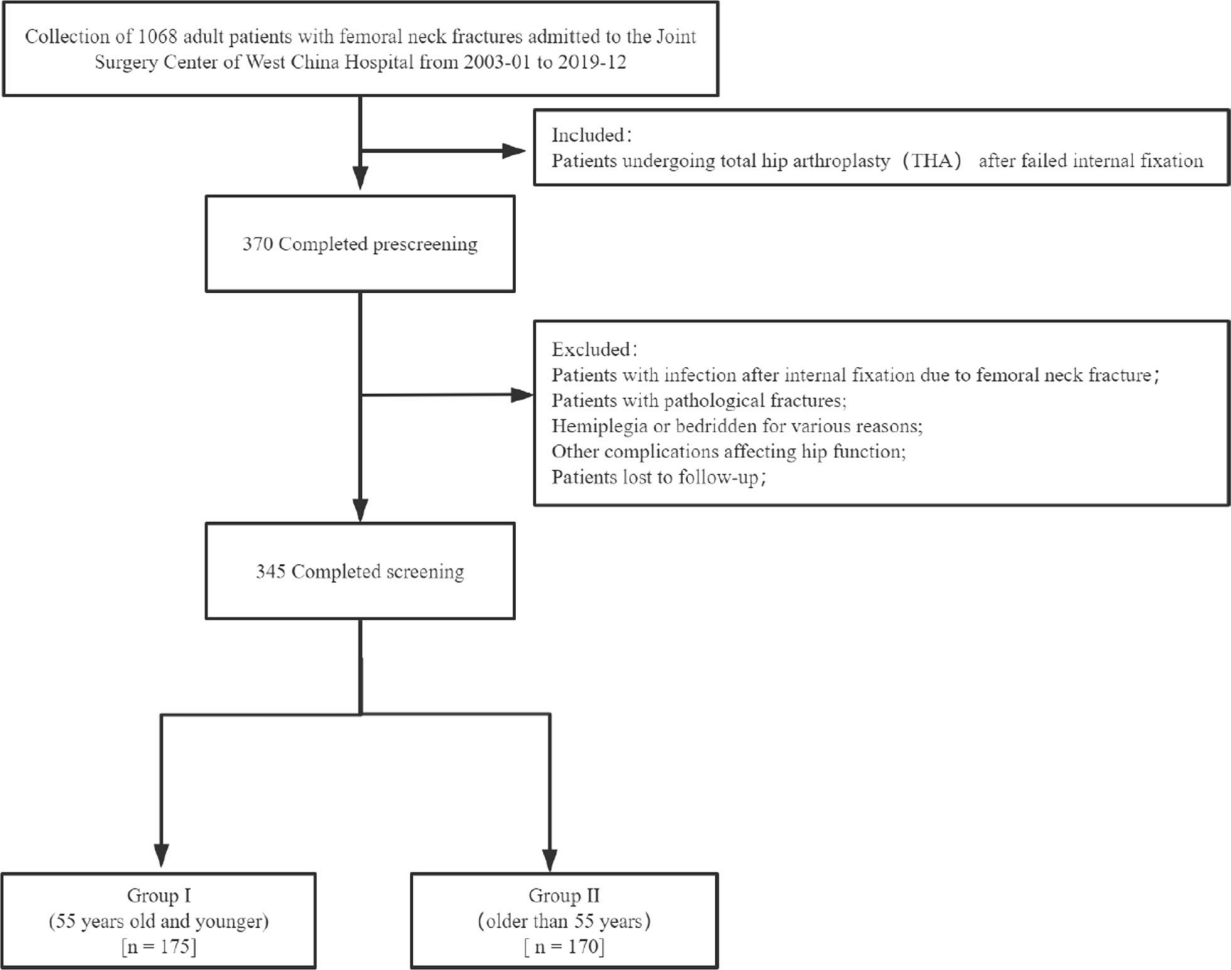
Flow diagram demonstrating methods to assess effect of salvage total hip arthroplasty after failed internal fixation of femoral neck fracture

Of the 1068 hospitalized patients with failed internal fixation of femoral neck fracture whom we screened for eligibility, 370 underwent THA. Of these, 11 were excluded because of infection after internal fixation, six because of pathological fracture or other complications affecting hip function, and eight because they were lost to follow-up. In the end, 345 patients (171 males) were included in the analysis, with mean age of 53.9 years (Fig. 2). The mean follow-up was 6 years. Younger patients (n = 175), defined as those no older than 55 years were defined as one group and compared to the remaining patients older than 55 years (n = 170). There was no significant difference in body mass index, other baseline clinicodemographic characteristics, or follow-up time between the two groups (Table 2).

**Table 2:** Patient demographic characteristics

Characteristic	All patients	Younger patients (≤ 55 years)	Older patients (> 55 years)	*p*
Number of hips	345	175	170	
Age	53.9 ± 13.8*	43.1 ± 9.3	65.1 ± 7.2	< 0.001
Sex				0.008
Male	171 (49.6)	99 (56.6)	72 (42.4)	
Female	174 (50.4)	76 (43.4)	98 (57.6)	
BMI, kg/m^2^				0.250
< 18.5	22 (6.9)	15 (9.1)	7 (4.5)	0.091
18.5–24.9	225 (70.1)	109 (66.1)	116 (74.4)	0.246
25.0–29.9	67 (20.9)	36 (21.8)	31 (19.9)	0.583
≥ 30.0	7 (2.2)	5(3.0)	2 (1.3)	0.468
Follow-up duration, mo.	73.6 ± 34.4	72.3 ± 33.9	75.1 ± 35.0	0.455
Reason for THA*				0.003
Nonunion	48 (16.3)	24 (17.1)	24 (15.5)	0.914
Femoral head necrosis	210 (71.2)	105 (75.0)	105 (67.7)	0.737
Posttraumatic osteoarthritis	37 (12.5)	11 (7.9)	26 (16.8)	0.007
Comorbidities				0.180
Hypertension	63 (48.5)	10 (43.5)	53 (49)	< 0.001
Diabetes	26 (20)	5 (21.7)	21 (19.6)	< 0.001
Cerebrovascular disease	5 (3.8)	0 (0.00)	5 (4.7)	0.067
Cardiovascular disease	14 (10.8)	2 (8.7)	12 (11.2)	0.005
Venous thromboembolism	5 (3.8)	3 (13)	2 (1.9)	1.000
Pulmonary disease	7 (5.4)	0 (0.00)	7 (6.5)	0.020
Chronic kidney disease	10 (7.7)	3 (13)	7 (6.5)	0.313
Type of internal fixation*				0.077
Intramedullary nail	3 (0.9)	2 (1.1)	1 (0.6)	1.000
Dynamic hip screw	20 (5.8)	6 (3.4)	14 (8.2)	0.056
Cannulated screw	217 (62.9)	115 (65.7)	102 (60.0)	0.272
Anatomic plate	22 (6.4)	15 (8.6)	7 (4.1)	0.091

Data are presented as n (%) or mean ± standard deviation, unless otherwise noted. *THA* total hip arthroplasty

*Some patients were not counted because of missing data

**Fig. 3:**
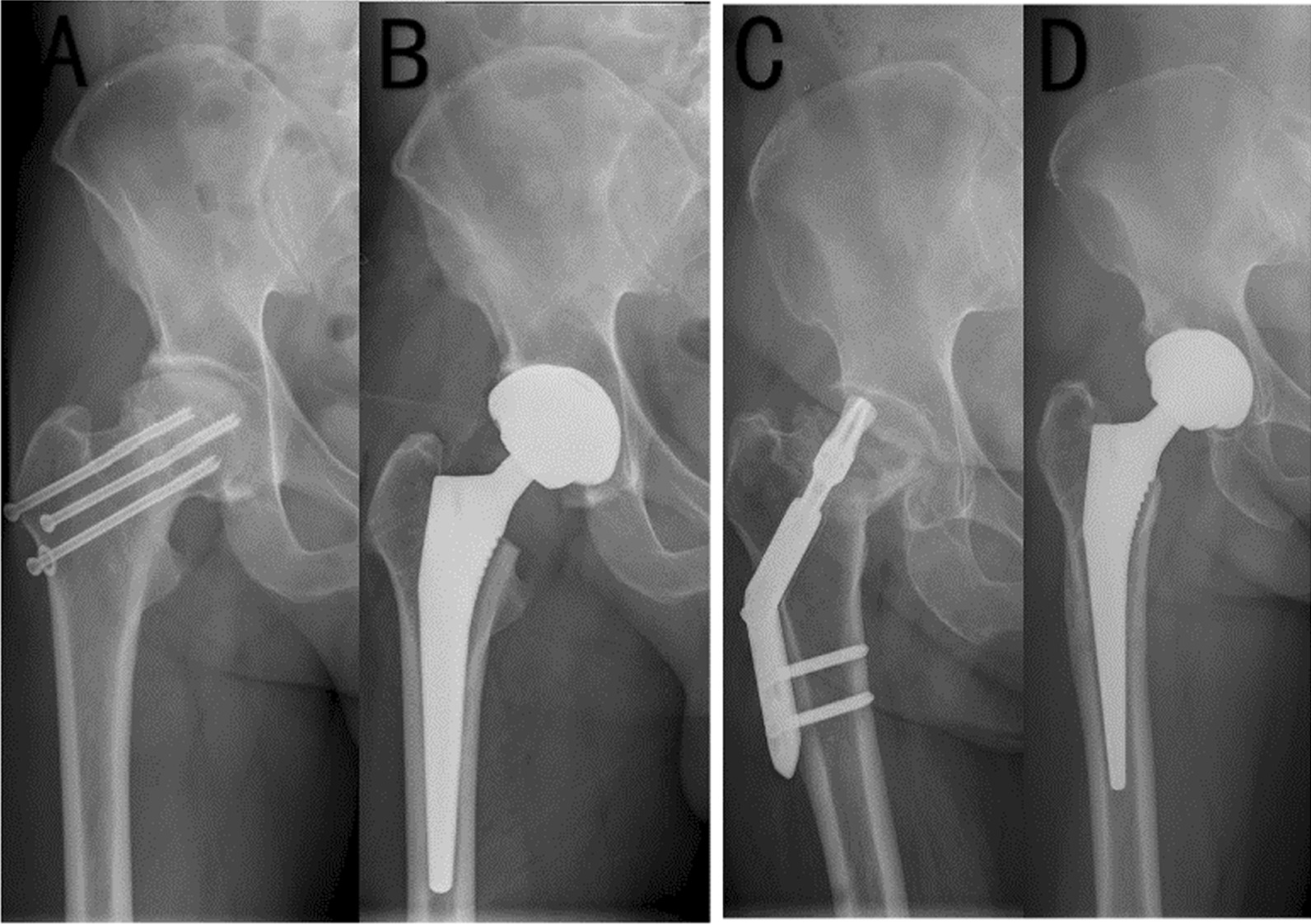
The imaging showing joint structure before and after operation. **A** Necrosis of femoral head after internal fixation of femoral fracture in Group I (anteroposterior film); **B** Total hip arthroplasty (THA) after internal fixation of femoral neck fracture in Group I (the last follow-up); **C** Necrosis of femoral head after internal fixation of femoral fracture in Group II (anteroposterior film); **D** THA after internal fixation of femoral neck fracture in Group II (the last follow-up)

Across all patients, HHS was significantly greater at last follow-up than preoperatively (89.2 ± 5.0 vs. 41.2 ± 6.1, p < 0.001, Table 3). HHS improved significantly in the separate groups of younger and older patients, but HHS at final follow-up was significantly higher in younger patients. The imaging before and after operation also showed joint structure after operation improved (Fig. 3). Younger patients were hospitalized for significantly shorter time than older patients (8.5 ± 4.1 vs. 9.5 ± 4.6 days, p = 0.036) and underwent THA significantly sooner after internal fixation (4.4 ± 4.7 vs. 6.8 ± 6.6 years, p < 0.001). The two groups did not differ significantly in intraoperative bleeding during THA.

**Table 3:** Perioperative indicators and Harris hip scores in patients after salvage THA

Indicator/variable	All patients (n = 345)	Younger patients (n = 175)	Older patients (n = 170)	*p*
Time between initial internal fixation and THA, years	5.5 ± 5.9	4.4 ± 4.7	6.8 ± 6.6	< 0.001
Blood loss, mL	188.6 ± 172.2	193.0 ± 171.9	183.5 ± 172.9	0.634
Length of hospital stay, days	9.0 ± 4.4	8.5 ± 4.1	9.5 ± 4.6	0.036
HHS				
Before THA	41.2 ± 6.1	39.9 ± 5.9	42.6 ± 6.0	< 0.001
At last follow-up	89.2 ± 5.0	90.2 ± 4.4	88.1 ± 5.3	< 0.001

Values are mean ± standard deviation, unless otherwise noted. *THA* total hip arthroplasty

**Fig. 4:**
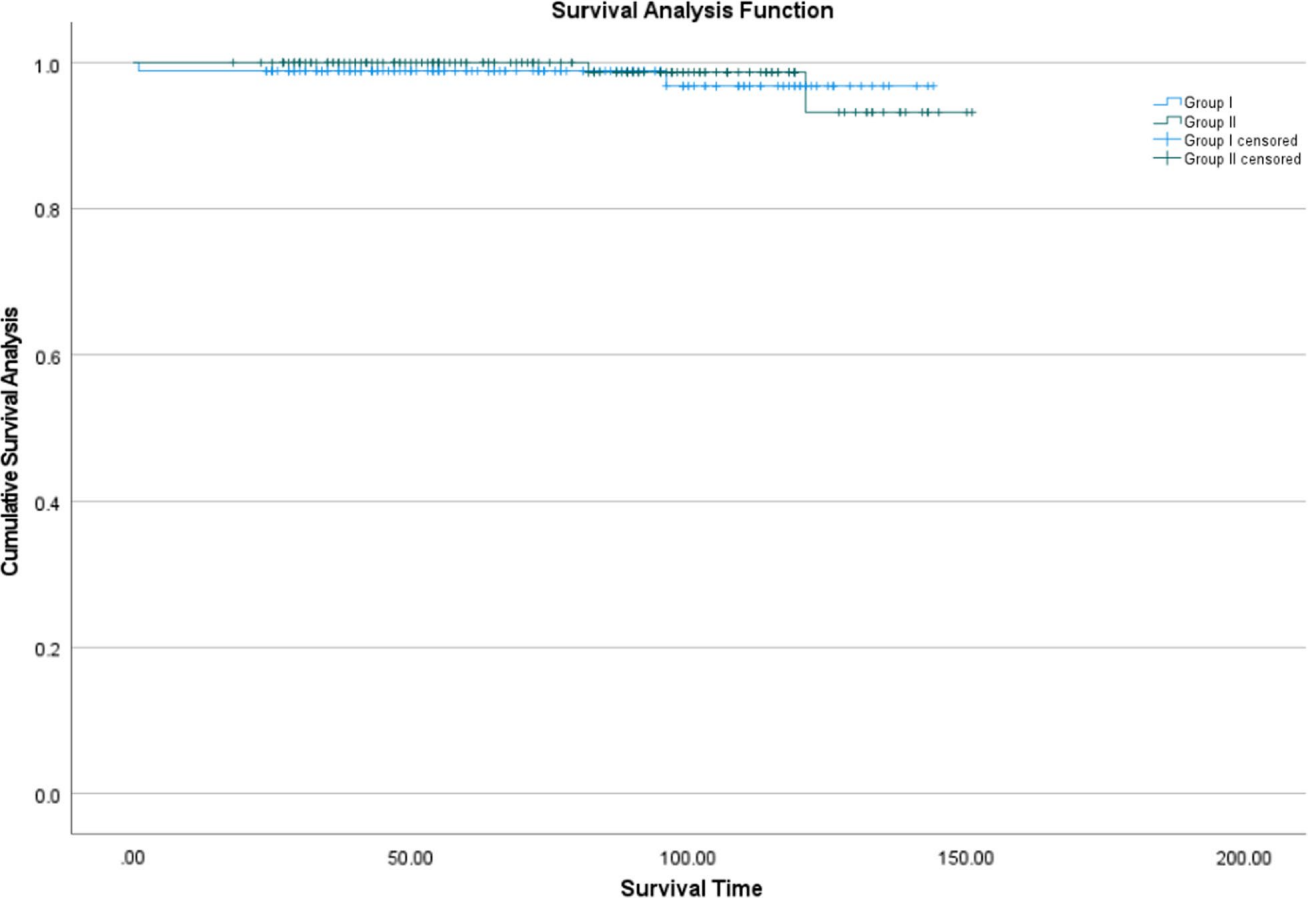
Kaplan–Meier survival free from revision surgery after THA in Group I and Group II. Group I stands for patients who are 55 years old and younger than 55 years old, and Group II stands for patients who are older than 55 years old

Across all patients, five experienced dislocation, three aseptic loosening, three surgical site infection, one prosthesis fracture, and two periprosthetic fracture (Table 4). The rate of postoperative complications was similar between the two groups. No death occurred within 1 year after surgery in either group. During follow-up, five patients (1.45%) needed revision surgery: three younger patients and two older ones. The 14-year survival rate did not differ significantly between the two groups (96.8 vs. 93.2%; p = 0.632; Fig. 4).

**Table 4:** Outcomes after salvage THA

Outcome	All patients (n = 345)	Younger patients (n = 175)	Older patients (n = 170)	*p*
Death within 1 year after surgery	0	0	0	–
THA-related readmission	13 (3.8)	7 (4.0)	6 (3.5)	0.818
Revision surgery for any reason	5 (1.4)	3 (1.7)	2 (1.2)	1.000
Complications				
Dislocation	5 (1.4)	4 (2.3)	1 (0.6)	0.385
Aseptic loosening	3 (0.9)	2 (1.1)	1 (0.6)	1.000
Operative site infection	3(0.9)	2 (1.1)	1 (0.6)	1.000
Prosthesis fracture	1 (0.3)	0 (0)	1 (0.6)	0.493
Periprosthetic fracture	2 (0.6)	1 (0.6)	1 (0.6)	1.000

Values are n (%), unless otherwise noted. *THA* total hip arthroplasty

## Discussion

In this retrospective cohort study, we investigated the mid- to long-term outcomes of salvage THA following failed internal fixation to treat femoral neck fracture. We also compared the outcomes between younger and older patients, given that few young patients have been included in previous studies of salvage THA. Our results suggest that salvage THA is well tolerated and can lead to good rates of postoperative survival without difference in different age groups. Our findings add to the growing body of evidence highlighting THA as a safe and effective rescue strategy in the event that internal fixation fails.

Whether age influences the outcomes of primary THA is controversial: some studies have concluded that it does not [23–25], while others have associated younger age with greater functional improvement [26–28]. A prospective, multicenter study of 7934 patients associated younger age (no older than 55 years) with significantly better functional scores following unilateral primary THA, but the difference was not clinically important [29]. Analogously, we found here that younger age was associated with higher HHS after salvage THA in younger patients than older ones, but the difference, while statistically significant, did not exceed the minimal clinically important difference [29]. Additional prospective, multicenter cohort studies are needed to assess the influence of age on outcomes of salvage THA after failed internal fixation.

We measured quite high survival without complications after salvage THA, similar to numerous previous studies of patients in whom internal fixation failed and who were followed up for similarly long periods as our cohort [30–35]. Our work substantially extends the literature by showing that such high survival and low complication rates apply to younger and older patients alike. In fact, complication-free survival after salvage THA in both age groups appears to be comparable to that in patients undergoing primary total hip replacement [36]. These results suggest that after internal fixation failure, salvage THA can replace the failed femoral neck, transfer stress, and substitute adequately for femoral neck function.

In theory, elderly patients would have significant physiological and psychological changes compared with young patients [37]. And the higher revision rate associated with non-cemented prosthesis is mainly due to the increased risk of early periprosthetic fractures, as reported [38]. However, in order to strictly control postoperative complications, our center follow up patients regularly, provide treatment contrapuntally and monitoring osteoporosis patients continuously. The incidence rate of periprosthetic fractures in older group is only 0.59%, which have no difference from the younger. Therefore, we speculate that strict complication-control may be the reason why we did not find a difference in survival rates between elderly and young patients. Further confirmed reasons require in-depth research and analysis next step.

The slightly higher incidence of some complications among younger than older patients in our cohort may reflect the generally higher physical activity among young people. For instance, 2.29% of our younger patients experienced dislocation, compared with only 0.59% of older patients. Younger age has been linked to greater risk of dislocation [39, 40]. Future studies should explore this age-dependent risk of dislocation, especially since more than 60% of patients who suffer one dislocation will suffer another, and over half will require revision surgery [41].

After the age of 55, there are significant physical and psychological changes for both men and women. Therefore, we believe that 55 years old is age juncture that can distinguish the outcomes of salvage total hip arthroplasty (THA). To the best of our knowledge, ours is the first study to analyze the efficacy of salvage THA in patients under 55 years after failed internal fixation of femoral neck fracture. At the same time, our study has some limitations. First, it involved retrospective analysis of patients from a single center. Second, our younger and older patients differed substantially in sex distribution and comorbidity burden, which may have confounded our analysis. Third, some patients in our study underwent salvage THA because of nonunion, post traumatic arthritis, and femoral head osteonecrosis, which involve different pathologies; this may have introduced heterogeneity and confounding into our analysis. Similarly, the multi-year lag between internal fixation and salvage THA may reflect the influence of age but also potentially numerous other variables, such as socioeconomic and lifestyle factors.

Despite these limitations, our study provides strong evidence that following the failure of internal fixation to treat femoral neck fracture, salvage THA can effectively improve function in younger and older patients, with low risk of complications in the mid- to long-term.
